# Endoscopic drainage for management of infected necrosis following EUS-TA in a patient with pancreatic cancer: A case report

**DOI:** 10.1097/MD.0000000000025466

**Published:** 2021-04-23

**Authors:** Young Jung Kim, Eunae Cho, Chang Hwan Park

**Affiliations:** Department of Gastroenterology and Hepatology, Chonnam National University Medical School, Gwangju, South Korea.

**Keywords:** endoscopic drainage, endoscopic ultrasound, infected pancreatic necrosis, pancreatic neoplasm

## Abstract

**Rationale:**

Endoscopic ultrasonography-guided tissue acquisition (EUS-TA) has become the norm for the diagnosis of pancreatic solid lesions. EUS-TA is relatively safe, but various complications can occur. Infected pancreatic necrosis (IPN) is a rare but serious complication. The latest guidelines suggest that all invasive interventions in patients with IPN should be delayed until walled-off necrosis appears.

**Patient concerns:**

A 73-year-old man was referred to our hospital with double primary cancers including gallbladder and pancreas. We performed EUS-TA on metastatic pancreatic tail cancer to confirm histologic diagnosis. Six days after the procedure, he developed abdominal pain and fever.

**Diagnoses:**

The patient's laboratory findings showed leukocytosis and C-reactive protein elevation. Fluid collection around pancreas tail and stomach was detected in computed tomography (CT) scan, and the patient was diagnosed with IPN.

**Interventions and outcomes:**

EUS-guided endoscopic transmural drainage (EUS-TD) was performed for the treatment of IPN. Two days after the procedure with antibiotics, his CRP level decreased abruptly, and he received chemotherapy for the treatment of pancreatic ductal adenocarcinoma (PDAC) 5 days after the procedure. He was discharged from our hospital without complications 15 days after chemotherapy.

**Lessons:**

In selected patients with PDAC, early endoscopic drainage may be recommended as treatment for IPN resulting from complications of EUS-TA.

## 1 Introduction

Endoscopic ultrasound-guided tissue acquisition (EUS-TA) has become the standard method for histologic diagnosis of pancreatic solid lesions because it is safe and efficient as well as indispensable.^[1]^ The general complication rate associated with EUS-TA has been stated to range between 1% and 2%.^[2]^ The major complications from EUS-TA include infections, bleeding, and pancreatitis along with gut perforation.^[3]^ Although the incidence of infection developing after EUS-TA is low, 1 potentially life-threatening complication is infected pancreatic necrosis (IPN).^[4]^ The latest guidelines recommend that all kinds of invasive interventions to treat IPN should be postponed until walled-off necrosis appears.^[5,6]^ However, early intervention for IPN following EUS-TA in pancreatic ductal adenocarcinoma (PDAC) can shorten the interval from the management of IPN to the curative treatment of PDAC. Herein, we report the case of early endoscopic drainage for the management of IPN induced by EUS-TA on PDAC.

## 2 Case report

**Figure 1:**
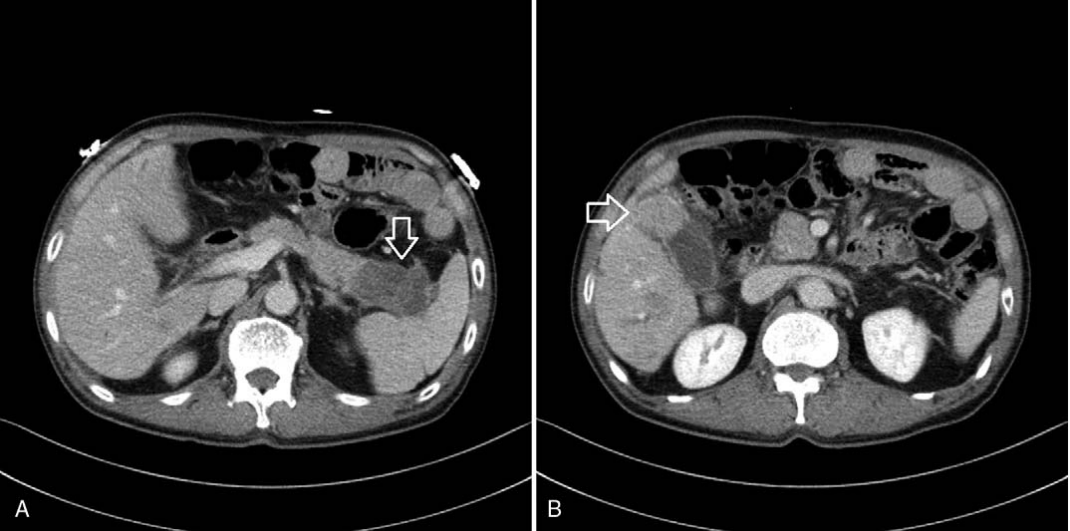
Abdominal CT. (A) A 63 × 37 mm heterogeneous enhancing low-attenuating mass in the tail of the pancreas. (B) A 40 × 35 mm heterogeneous enhancing mass in the gallbladder fundus (arrows). CT = computed tomography.

A 73-year-old man with suspected double primary cancers including gallbladder and pancreas was referred from a local medical center. He had a body weight loss of about 9 kg over 2 months, 58 kg at admission compare to a previous weight of 67 kg. He denied fever with chills and had no abdominal pain. He showed increases in levels of tumor markers: carcinoembryonic antigen 60.35 ng/mL and carbohydrate antigen 19-9 > 12,000 U/mL. On the contrast-enhanced abdominal computed tomography (CT) scan from the local medical center, a low-attenuating mass of about 63 × 37 mm in size was suspicious in the tail of the pancreas (Fig. 1A) and a 40 × 35 mm sized enhancing mass was noted in gallbladder fundus (Fig. 1B).

The patient had a history of distal gastrectomy with Billroth I anastomosis for the treatment of gastric cancer 27 years ago. He had been taking medications for type 2 diabetes mellitus. He reported consuming 3 bottles of alcoholic beverages per week and quit smoking 30 years ago.

**Figure 2:**
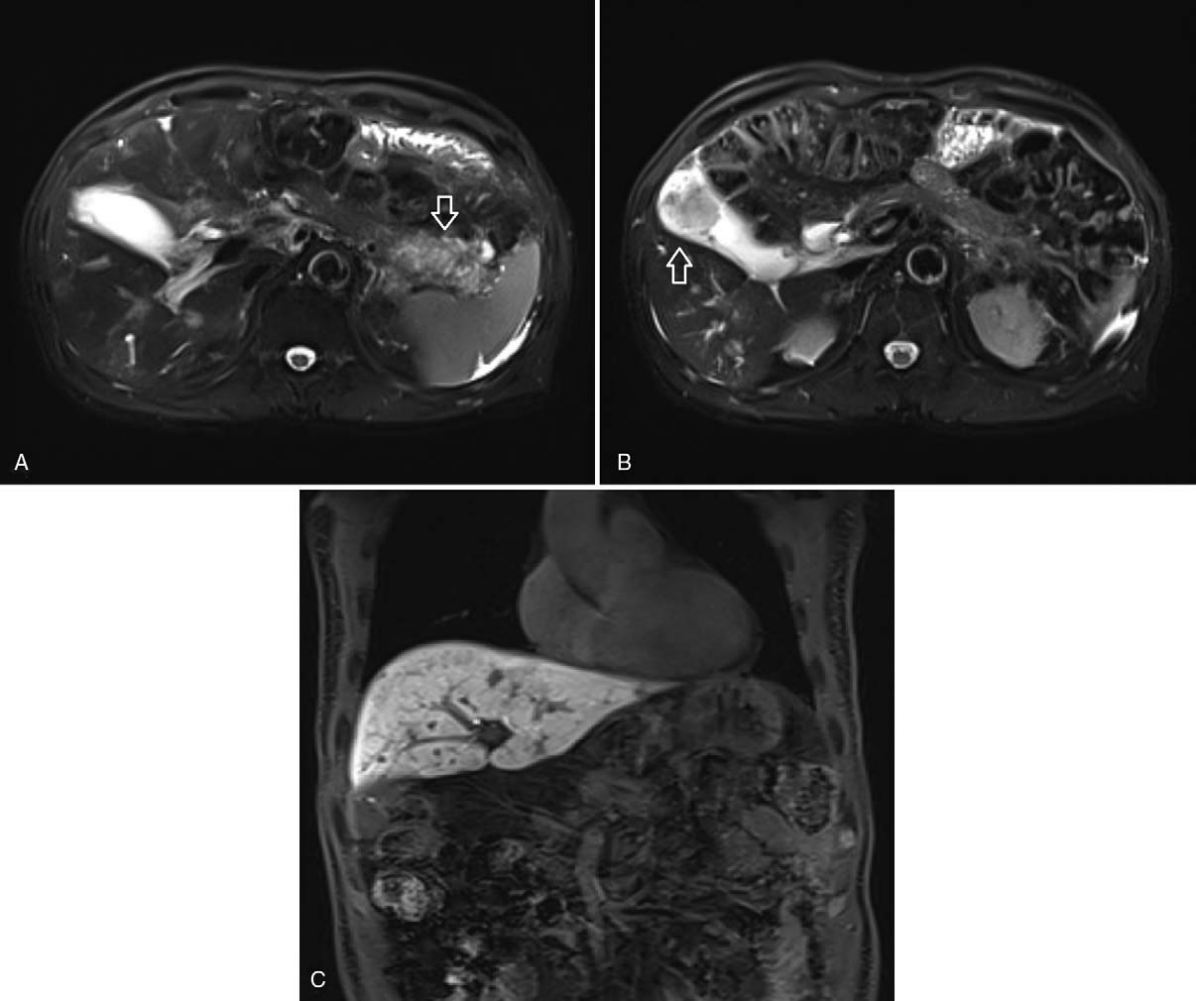
Pancreas MRI. (A) A 62 × 40 mm delayed enhancing mass in the tail of the pancreas with splenic vessel invasion (arrows). (B) A 37 × 34 mm heterogeneously enhancing mass in the gallbladder (arrows). (C) Multiple hepatic metastases. MRI = magnetic resonance imaging.

**Figure 3:**
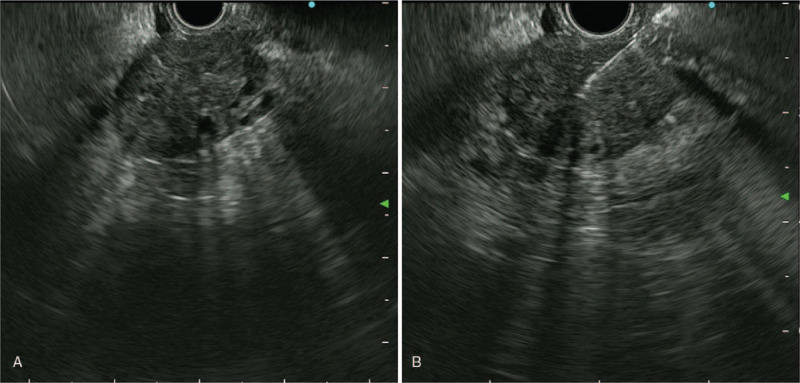
EUS. (A) A 35 mm heterogeneous hypoechoic lesion with anechoic fluid collection in the pancreas tail. (B) Necrotic tissues were aspirated from the pancreatic lesion by EUS-TA. EUS = endoscopic ultrasound, EUS-TA = endoscopic ultrasound-guided tissue acquisition.

On the admission day, magnetic resonance imaging was performed to further characterize the pancreatic and gallbladder lesions. On magnetic resonance imaging (MRI), a 62 × 40 mm sized mass was seen in the tail of the pancreas with splenic vessel invasion and multiple hepatic metastases (Fig. 2A–C). EUS-TA with a 22-gauge needle (EZ Shot; Olympus Co., Tokyo, Japan) was performed on metastatic pancreatic tail cancer to confirm histologic diagnosis. EUS (GF-UCT260; Olympus Co., Tokyo, Japan) showed a 35 mm sized hypoechoic heterogeneous lesion in the pancreas tail (Fig. 3A). Target tissue was obtained from the pancreatic lesion through EUS-TA (Fig. 3B). Two days later, the lesion was confirmed as PDAC according to histologic findings.

Six days after EUS-TA, abdominal pain developed with a fever (body temperature of 37.9°C). He demonstrated mild abdominal distension with direct tenderness in the epigastric area. The laboratory findings were as follows: white blood cell count 7,800/μL (neutrophil 78.7%), hemoglobin 11.3 g/dL, platelet count 106,000/μL, aspartic acid aminotransferase 45 IU/L, alanine aminotransferase 34 IU/L, total bilirubin 1.5 mg/dL, blood urea nitrogen 12.7 mg/dL, creatinine 0.96 mg/dL, C-reactive protein 16.25 mg/dL, fasting serum glucose 193 mg/dL, and hemoglobin A1c 7.0%.

**Figure 4:**
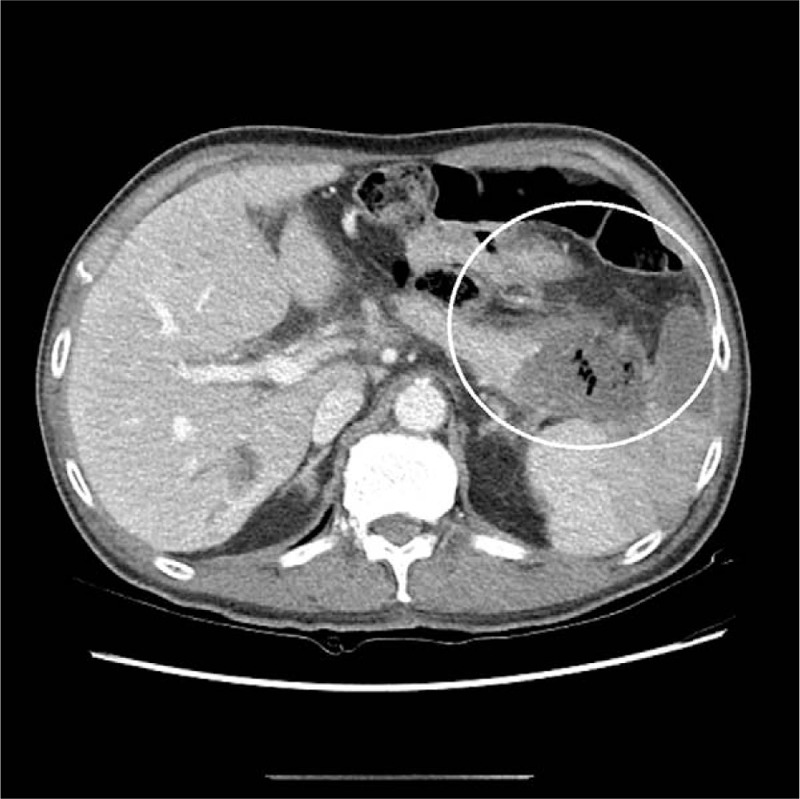
Abdominal CT. A marked fluid collection between pancreatic tail and stomach (white circle). CT = computed tomography.

Abdominal CT showed marked fluid collection between pancreatic tail and stomach (Fig. 4). Infected necrosis was suspected on the basis of his symptoms, laboratory findings, and abdominal CT. Empiric antibiotics such as intravenous ceftriaxone and metronidazole were started.

The next day, fever persisted, and some laboratory findings were aggravated: white blood cell count 11,800/μL (neutrophil 77.5%), aspartic acid aminotransferase 90 IU/L, alanine aminotransferase 72 IU/L, total bilirubin 1.92 mg/dL, and C-reactive protein 18.57 mg/dL.

**Figure 5:**
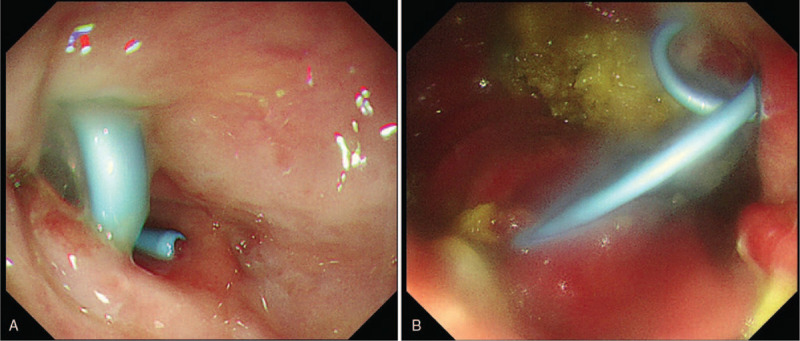
Purulent material flowed via the stent into the gastric body following deployment (A–B).

**Figure 6:**
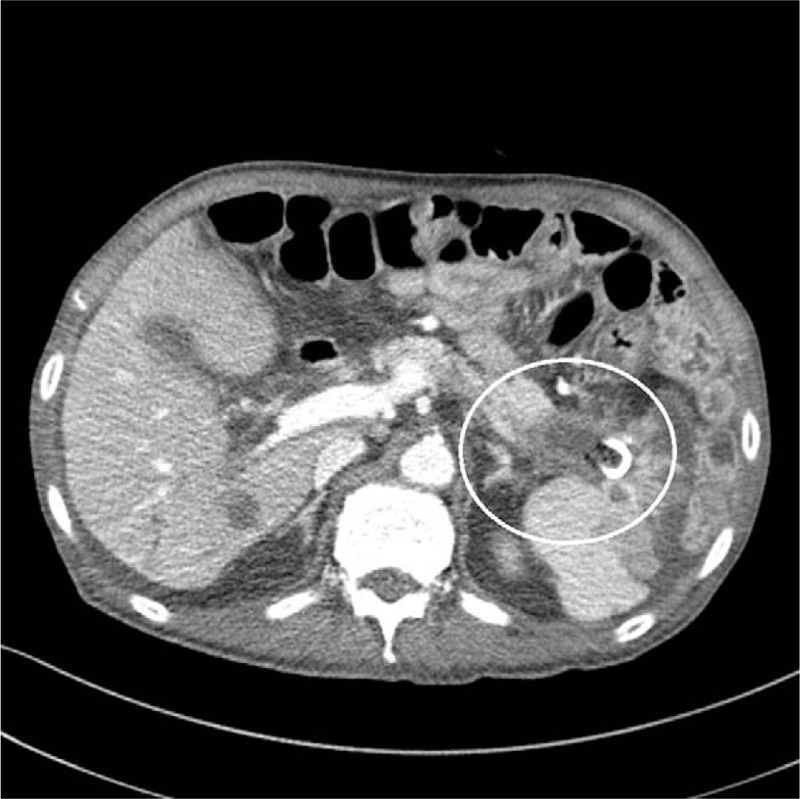
Abdominal CT shows the decrease from 63 to 41 mm in the mass (white circle) in the pancreatic tail. CT = computed tomography.

EUS-guided transmural drainage (EUS-TD) was performed for the treatment of infected necrosis (Fig. 5A–B). We did not perform a culture of necrotic material drained. There were no adverse events associated with the procedure. The general condition and laboratory findings of the patient rapidly improved starting the day after the procedure. Five days after the procedure, he was able to receive chemotherapy with FOLFIRINOX (a four-drug combination of 5-FU, leucovorin, oxaliplatin, and irinotecan). A subsequent abdominal CT at 2 weeks after EUS-TD proved a reduction in the size of infected necrosis from 63 mm to 41 mm (Fig. 6). The patient was discharged with no further complications.

## 3 Discussion

Complications associated with EUS-TA are rare, but they worsen the prognosis of patients with PDAC if the appropriate treatment is delayed. The complication risk for EUS-TA on pancreatic masses has been reported to be like that of upper endoscopy.^[7–9]^ The major complications are bleeding, acute pancreatitis, gut perforation, and infection.^[3,10]^ Among them, the risk of infection is low and routine antibiotic prophylaxis is not recommended before EUS-TA of solid pancreatic lesions.^[11–13]^ EUS-TA of pancreatic cystic lesions has been known to increase the risk of bacteremia so that prophylactic antibiotics have been recommended.^[14]^ However, a recent randomized trial and meta-analysis of 40 studies reported that the incidence of cyst infection after EUS-TA was less than 1%. Prophylactic antibiotics are therefore no longer needed before EUS-TA of pancreatic solid and cystic lesions.^[15,16]^

PDAC is an aggressive malignancy with a high mortality rate. Currently, the only potentially curative option for PDAC is surgical resection with negative margins, but only a small number of patients have operable tumors.^[17]^ The characteristics of PDAC include fast growth and rapid metastasis to other organs. Early detection of PDAC is a key factor in increasing the number of patients eligible for surgery. In addition to early detection, once PDAC is diagnosed, it requires prompt treatment. Delayed treatment could cause an originally resectable tumor to become an unresectable one. PDAC is frequently accompanied by infection, biliary obstruction, and pancreatic insufficiency. Beyond these factors, the hypercatabolic state of cachexia and muscle loss are prevalent among PDAC patients.^[18]^ For these reasons, the overall condition of patients with PDAC tends to deteriorate rapidly and contributes to decreased tolerance for systemic treatments. Drug regimens such as FOLFIRINOX for PDAC are suitable only for those patients with good performance status due to their significant toxicity.^[19]^ Delayed treatments could worsen the overall condition of PDAC patients, and many in this population may not be eligible for chemotherapy. Thus, patients with PDAC are recommended for prompt treatment avoiding delay to the greatest extent possible.

According to the revised 2012 Atlanta classification of acute pancreatitis, the complication of necrotizing pancreatitis is categorized as early and late. The mortality rate of sterile necrotizing pancreatitis was reported to less than 10% whereas if necrosis becomes infected, the mortality rate increases to 20% to 30%.^[20]^ Thus, early recognition of IPN and initiation of appropriate therapy is necessary. Current guidelines recommend delay of all forms of invasive interventions in patients with IPN, preferably until the necrotic collection forms wall-off and the liquid component predominates.^[5,6]^ Some PDACs undergo central necrosis and form the necrotic collection.^[21]^ In necrotic collection, infection could occur as a complication associated with EUS-TA. If the infected necrotic collection were sufficiently liquefied, it would be possible to drain the lesion as an effective treatment. Early treatment of infected necrosis could lead to surgery or chemotherapy for PDAC at an appropriate time without delay.

There are several limitations in attempting early endoscopic drainage of the infected necrosis that occurs as complication with EUS-TA in patients with PDAC. First, needle tract seeding, although uncommon, can occur after EUS-TA, and this complication seriously affects the prognosis of patients. Since the first report in 2003, needle tract seeding associated with EUS-TA has been reported in 15 cases.^[22]^ In the case of endoscopic drainage, the probability of tumor seeding is estimated to be higher than that of diagnostic fine needle aspiration, considering the formation of a fistula tract and longer procedure time. The current case is an inoperable pancreatic cancer with multiple metastases, and the benefit of the EUS-TD was greater than the risk of tumor seeding related to the procedure. However, when performing EUS-TD in resectable tumors, the possibility of needle tract seeding should be considered. The presence of infected necrosis at technically challenging locations is another limitation of EUS-TD.^[23]^ In this case, infected necrosis was in the pancreatic tail, and the lesion was easily accessible via the gastric body. However, if the lesion is in a technically inaccessible area, conservative treatment may be recommended considering the high risk of the procedure.

Infected necrosis associated with EUS-TA for PDAC is a rare but potentially life-threatening complication. It delays proper treatment for PDAC, which adversely affects the outcome of the disease. Therefore, it is necessary to perform appropriate treatment early. However, the standard guideline has not yet been established. In this case, we performed early endoscopic drainage of the infected necrosis, and it showed a good prognosis. In selected PDAC patients, early EUS-TD may serve as a recommendable treatment for infected necrosis. Additional cases should be accumulated to determine the overall effect of early endoscopic drainage on infected necrosis in patients with PDAC.

## Author contributions

**Conceptualization:** Chang Hwan Park.

**Methodology:** Young Jung Kim, Eunae Cho.

**Project administration:** Young Jung Kim, Eunae Cho.

**Resources:** Young Jung Kim, Eunae Cho, Chang Hwan Park.

**Writing – original draft:** Young Jung Kim.

**Writing – review & editing:** Young Jung Kim, Chang Hwan Park.
